# The possibilities of conscious experience in light of the dual origin hypothesis of the neocortex

**DOI:** 10.1093/nc/niaf058

**Published:** 2026-01-04

**Authors:** Lorena Chanes, Miguel Ángel García-Cabezas

**Affiliations:** Departament de Psicologia Clínica i de la Salut, Universitat Autònoma de Barcelona, 08193 Bellaterra (Cerdanyola del Vallès), Barcelona, Spain; Institut de Neurociències, Universitat Autònoma de Barcelona, 08193 Bellaterra (Cerdanyola del Vallès), Barcelona, Spain; Departmento de Anatomía, Histología y Neurociencia, Facultad de Medicina, Universidad Autónoma de Madrid, calle Arzobispo Morcillo 4, 28029 Madrid, Spain; Neural Systems Laboratory, Department of Health Sciences, College of Health and Rehabilitation Sciences, Boston University, 635 Commonwealth Avenue, Boston, MA 02215, United States

**Keywords:** conscious experience, cortical organization, predictive processing, dual origin hypothesis

## Abstract

According to contemporary psycho- and physiological perspectives, the brain supports our experience of the world by constantly anticipating what may happen next. In this context, limbic mesocortical areas have been proposed to play a key domain-general role in cortical processing, holding highly abstract content that may be efficiently broadcasted to virtually the whole brain, ultimately integrating interoception into a unified field of experience from the point of view of someone who has a body. Here we ground the evolutionary basis of such structural and functional organization in the hypothesis of the dual origin of the neocortex, suggesting that the addition of phylogenetically newer cortical types with modality-specific processing may have enabled the primitive polysensory role of limbic mesocortical areas to evolve into a multimodal coordinator within an ever more complex brain, favoring the possibilities of conscious experience. Moreover, two fundamental functional axes with relevance for allostasis emerge: (i) a navigation/spatial versus exchange/contact axis; and (ii) a sensing versus acting axis. The former summarizes a fundamental distinction between spatial navigation and musculoskeletal control versus close interactions in the intimate and internal spheres; the latter reflects a functional (although intimately linked) distinction between sensory and motor aspects. These axes define a conceptual bidimensional space across cortical types where virtually all cortical areas may be placed according to their functional relevance, with limbic mesocortices ultimately integrating experience across sensory-motor function and navigation-exchange. These notions have important implications for our understanding of allostasis and human experience.

## Introduction

Limbic mesocortical areas have been proposed to play a key domain-general role in cortical processing (Chanes and Barrett 2016, Barrett 2017). These areas, which are cytoarchitectonically defined as agranular and dysgranular (Barbas 2015, García-Cabezas et al. 2020), and also referred to as paralimbic (Mesulam 1998), would hold highly abstract content that could be efficiently accessed by virtually the entire brain. This notion emerged from the implementation of predictive processing perspectives of brain function at the level of neural circuits applying the principles of the structural model of cortico-cortical connections. The direct consequence of this implementation was that limbic mesocortical areas occupied high levels of the hierarchy across cortical systems, possibly functioning as a “workspace”, broadcasting predictions to lower-level more specialized areas, with important implications for allostasis, conscious experience, and brain-related conditions (Chanes and Barrett 2016, Barrett 2017, Chanes and Barrett 2020; see also Barrett and Simmons 2015 for seminal work regarding interoception). More recent views of brain function based on predictive processing have further emphasized allostasis, proposing that our experiences emerge within a general role of the brain to predictively ensure its maintenance (Barrett 2017), which in turn is closely linked to brain organization (Katsumi et al. 2022, Katsumi et al. 2023).

In recent years, theoretical frameworks focusing on domain-general principles of cortical development, organization, and function, such as the structural model and the limbic workspace model among others, have contributed to a progressive shift from the classical construct of cortical area to the concepts of cortical gradients and cortical types, with the potential to integrate knowledge in a coherent view, from developmental biology studies in vertebrate embryos to *in vivo* neuroimaging studies in humans and behavior (for recent examples, see, e.g. Margulies et al. 2016, Paquola et al. 2022, Aparicio-Rodríguez and García-Cabezas 2023, Katsumi et al. 2023, Ruiz-Cabrera et al. 2023, Saberi et al. 2023, King and Weiner 2024, Ohm et al. 2024, Sebenius et al. 2024, Paquola et al. 2025). In this article, we analyze the integration of predictive processing approaches within the neuroanatomical notions of cortical gradient and cortical type, further grounding it within the evolutionary expansion of the primate cerebral cortex, with significant implications for our understanding of allostasis and how we experience the world. More specifically, we used the hypothesis of the dual origin of the neocortex, proposed by Friedrich Sanides (1962, 1970), which is supported by contemporary developmental data (Medina et al. 2004, Abellan et al. 2014, Gonzalez-Gomez and Meyer 2014, Siskos et al. 2021). The hypothesis of Sanides provides an explanation for the tangential expansion of the cerebral cortex during evolution by the addition of novel cortical types in concentric rings that evolved from two ancestral allocortical sectors that formed the oldest and most outer cortical ring: the ancestral hippocampal formation and the ancestral olfactory cortex. The development and expansion of the cortex starting at this phylogenetically ancient cortical ring would have been a key step in the emergence of the limbic workspace and complex forms of cognition. Moreover, two key functional axes emerge across cortical types. The first one, which could be traced to the two ancestral sectors of the allocortical ring, refers to the navigation versus exchange distinction, emphasizing the spatial and navigational aspect of experiencing the world versus the intimate exchange and contact. The second one would be a sensory versus motor axis, reflecting a fundamental functional (although intimately related) distinction between sensing and acting.

## Limbic mesocortical areas within the general principles of cortical organization

**Figure 1:**
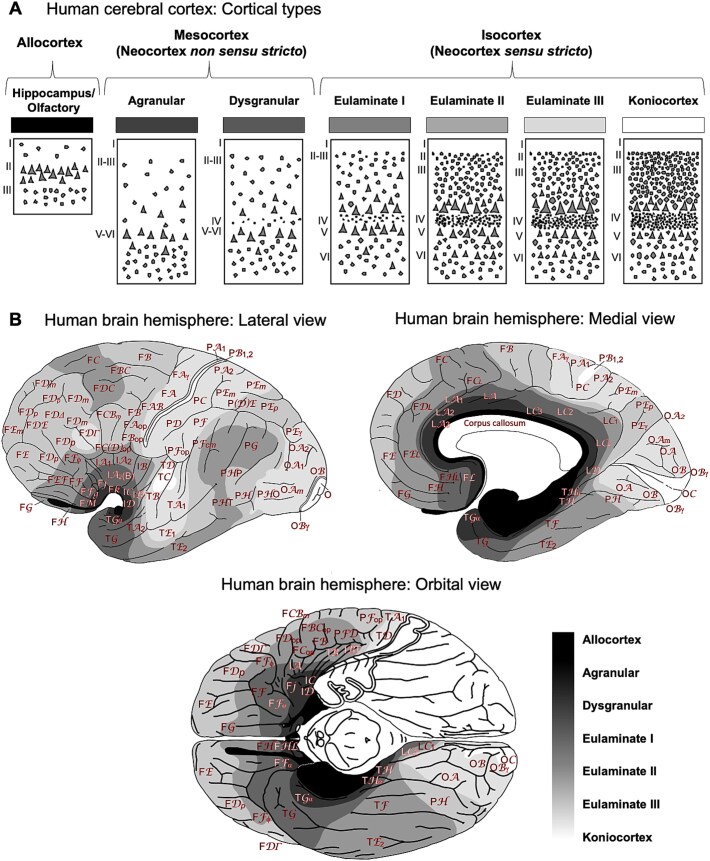
Cortical types in the human cerebral cortex. (A) Sketches of cortical types from the simplest (left) to the most complex (right) types [modified from Uceda-Heras et al. (2024)]. (B) Maps of the human cerebral cortex depicting the distribution of cortical types in greyscale [black: simplest type; white: most complex type; modified from García-Cabezas et al. (2020)].

The limbic workspace notion (Chanes and Barrett 2016) emerged when integrating a predictive processing view of how the brain works (Friston 2005, 2010, Clark 2013), which emphasizes the relevance of anticipation on how we experience the world, with a neuroanatomical model of synaptic cortico-cortical connections, known as the structural model (Barbas and Rempel-Clower 1997, García-Cabezas et al. 2019). This model highlights the relevance of cortical types for cortical organization (García-Cabezas et al. 2020). Cortical types are operationally defined along a gradient of laminar complexity that starts adjacent to the allocortex and progresses into the neocortex, from limbic mesocortical areas (neocortex *non sensu stricto*) into progressively more complex isocortical areas (neocortex *sensu stricto*), culminating in koniocortices (Fig. 1). Importantly, the structural model relates cortical types to the direction of information flow along the cerebral cortex. More specifically, feedback (top-down) connections originate in cortical areas with a simpler laminar architecture and feedforward (bottom-up) connections originate in areas with a more complex laminar architecture (Barbas and Rempel-Clower 1997, García-Cabezas et al. 2019). In predictive processing terms, limbic mesocortical areas, being the neocortical areas with the simplest laminar architecture (agranular and dysgranular), are at the top of the predictive neocortical hierarchy, issuing predictions (top-down) to the rest of the neocortex as efficient summaries that become more specific as better laminated, more specialized, areas are reached (Chanes and Barrett 2016).

Adding to their privileged anatomical position occupying the top of neocortical hierarchies, limbic mesocortical areas are strongly interconnected and have strong reciprocal connections with the allocortex [hippocampal formation (Barbas and Blatt 1995) and primary olfactory cortex (Carmichael et al. 1994)], the amygdala (Ghashghaei et al. 2007), the hypothalamus (Rempel-Clower and Barbas 1998), and key brainstem structures such as the periaqueductal gray (An et al. 1998). Taken together, this suggests that limbic mesocortical areas may function as a workspace for conscious experience, holding highly abstract (general) information that can be efficiently broadcasted to virtually the entire brain. This “workspace” would support (may not be sufficient for) conscious experience, particularly by enabling highly general, abstract, and allostatically relevant information, and by dynamically interacting with multiple functional cortical and subcortical systems across the brain. The “broadcasting” notion evokes the global neuronal workspace hypothesis, which proposes that, “in a conscious state, a non-linear network ignition associated with recurrent processing amplifies and sustains a neural representation, allowing the corresponding information to be globally accessed by local processors” (Mashour et al. 2020). Unlike the global neuronal workspace model (Dehaene and Changeux 2011), which emphasizes large-scale fronto-parietal circuits involved in conscious access and widespread broadcast, the limbic workspace was explicitly grounded in the gradient of laminar complexity of the primate cortex and its relationship to the laminar patterns of cortico-cortical synaptic connections, as stated by the structural model. According to this model, whose principles may be extrapolated to the human cortex (Uceda-Heras et al. 2024, Barbas et al. 2025), mesocortical limbic areas are the ultimate feedback-sender of the neocortex and, therefore, are at the top of neocortical hierarchies. Thus, the limbic workspace posits a key role for mesocortical limbic areas in cortical processing and emphasizes these regions’ participation in broadcasting *allostatically* integrated predictions across the cortex, enabling ongoing, context-sensitive integration of affective and motivational relevance within hierarchical predictive processing architectures.

The relevance of the cortical gradient and the limbic workspace constructs is supported by a body of literature across the brain structural and functional domains. The limbic end of the laminar gradient, i.e. mesocortical areas, has been related to slower timescales (Kiebel et al. 2008, Murray et al. 2014) and is topologically central when considering the cerebral cortex as a network (Zhang et al. 2020), consistent with these areas dealing with a higher level of abstraction and a relatively stable internal model. Evidence from network control theory is also consistent with a limbic workspace being at the apex of a sensory-fugal axis (Parkes et al. 2022). Moreover, postmortem studies have demonstrated that the expression of markers that favor synaptic plasticity and epigenetic regulation is higher in limbic meso- than in isocortical areas (García-Cabezas et al. 2017, Sancha-Velasco et al. 2023), which could possibly render limbic mesocortical areas vulnerable to abnormal structural changes, as seen in brain-related conditions. Indeed, in neurodegenerative diseases, the aggregation and dissemination of pathological tau follows a specific temporospatial spreading pattern and propagates from limbic mesocortical to progressively better laminated isocortical areas (Ohm et al. 2024, Ottoy et al. 2024, Uceda-Heras et al. 2024, Barbas et al. 2025). With regards to mental health-related conditions, in autism, for example, recent research (Weber et al. 2024) confirmed *in vivo* what had previously been observed in postmortem studies (Zikopoulos and Barbas 2010) and discussed in theoretical interpretations based on the limbic workspace model (Chanes and Barrett 2020). These interpretations highlighted domain-general disruptions of connectivity at high levels of the cortical hierarchy, notably in mesocortical areas, limiting integration and abstraction (Chanes and Barrett 2020, Weber et al. 2024). Similarly, the limbic workspace model may function as a common framework to interpret the impact of focal brain lesions, as done in a seminal paper by Giaccio (2006) revealing that lesions toward the limbic mesocortical end of the laminar gradient lead to disturbances of core aspects of a person’s experience of/in the world, in contrast with the more focal impairments of motility and volition observed when the damaged areas have a more complex laminar structure, something that is also observed across psychiatric diseases (Park et al. 2022). Together, this evidence supports the notion that limbic mesocortical areas occupy high levels in the cortical hierarchy, holding abstract representations, and yielding predictions that can reach the whole brain, with fundamental consequences for a person’s experience of the world.

At the microcircuit level and consistent with the structural model, superficial layers convey prediction errors while deep layers convey predictions (Bastos et al. 2012). Although these microcircuits are considered canonical and applicable to the entire neocortex, it needs to be noted that, in parallel to laminar architecture, several cellular and molecular features vary gradually, providing each cortical type with different computational capacities that may differentially support predictive processing. For instance, dendritic arbors of cortical projection neurons are not homogeneous along the cerebral cortex: the extension of basolateral dendrites in layer III pyramidal neurons is smallest in koniocortices, increasing progressively through isocortical areas, and is largest in mesocortical areas of macaques (see Table 2 in García-Cabezas et al. 2018); this suggests that the dendritic arborization of pyramidal neurons, which is related to their receptive field, increases from the konio- toward the mesocortices, potentially impacting how predictions are received. Other cellular and molecular factors that contribute to fine circuit processing, like myelin and the strongly inhibitory parvalbumin interneurons, also decrease gradually from the konio- through iso- into the mesocortices (García-Cabezas et al. 2017, Zikopoulos et al. 2018, Sancha-Velasco et al. 2023). In opposite direction, factors that favor synaptic plasticity and epigenetic regulation decrease from meso- through iso- into the koniocortices (García-Cabezas et al. 2017, Sancha-Velasco et al. 2023). The variation of all these cellular and molecular features suggests that mesocortical neurons are more plastic and less stable, as mentioned above, and may integrate more information in their larger dendritic fields, consistent with a more generalized role in cortical processing. In contrast, neurons in areas at the other end of the gradient would be less plastic, more stable, and have smaller receptive fields, thus supporting a more specialized processing. Thus, the systematic variation of all these features may endow mesocortical agranular areas with the capacity to generate broad, top-down predictions, while eulaminate sensory cortices, which are more differentiated, may be optimized to convey specialized bottom-up prediction errors.

## Grounding the phylogenetic basis within the hypothesis of the dual origin of the neocortex

**Figure 2:**
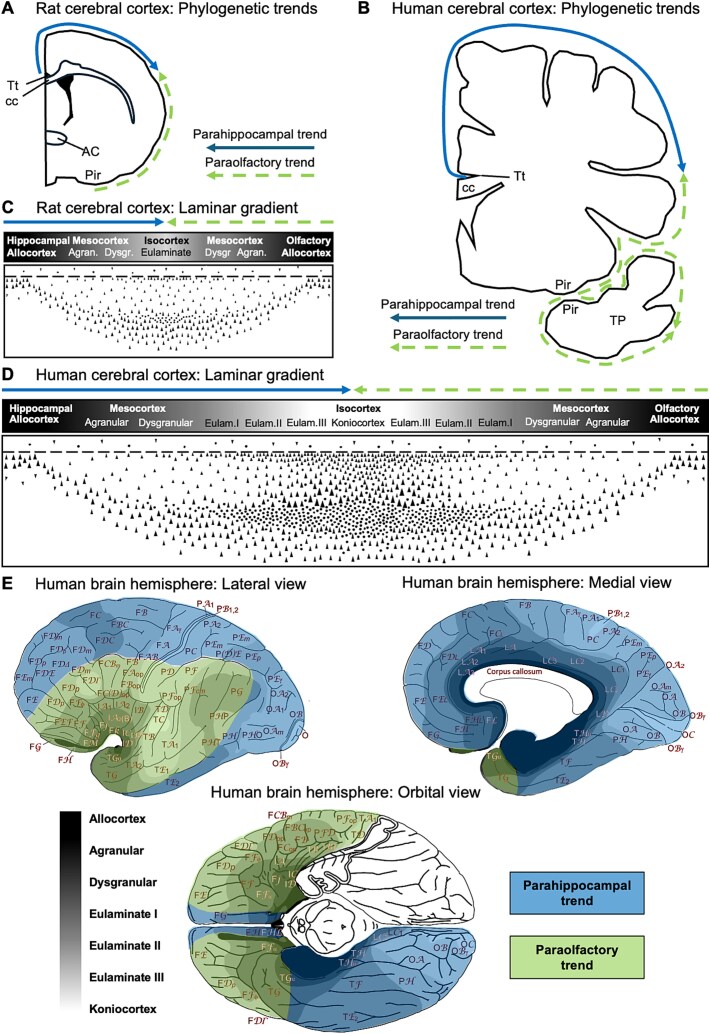
Cortical phylogenetic trends according to the hypothesis dual origin of the neocortex. (A) and (B) Parahippocampal (blue solid arrow) and paraolfactory (green dashed arrow) trends in a coronal section of the rat (A) and human (B) brain. (C) and (D) Cortical types and parahippocampal (blue solid arrow) and paraolfactory (green dashed arrow) trends along the gradient of laminar complexity in the rat (C) and human (D) cerebral cortex. (E) Maps of the human cerebral cortex depicting the distribution of cortical types in greyscale (black: simplest type; white: most complex type); the areas within the parahippocampal and the paraolfactory trends are shadowed in blue and green, respectively. The boundary between the two trends is delineated roughly by the inferior frontal sulcus and the intraparietal sulcus (Sanides 1970). Most of the orbitofrontal cortex is a derivative of the paraolfactory trend: the most posterior portion of the central and lateral parts of the orbitofrontal cortex are adjacent to the olfactory allocortex (anterior olfactory nucleus and the piriform cortex). However, we have considered its most medial part, the straight gyrus, as parahipocampal because it has a clear topological relation with the hippocampal allocortex. It must be noted that the two halves of the allocortex (the hippocampal and olfactory allocortices) form a closed ring. The anterior extension of the hippocampal formation (tenia tecta) contacts the anterior olfactory nucleus at the most posterior part of the orbitofrontal cortex. Thus, hippocampal allocortex can be found along the entire extension of the corpus callosum giving rise to the cingulate mesocortices and their adjacent isocortices along the entire medial face of the brain hemisphere. In primates, the gyrus rectus is adjacent to the tenia tecta, which is an anterior extension of the hippocampal formation (see Fig 3 in Sanides 1970 and the cytoarchitectonic sequence from hippocampal allocortex to eulaminate I isocortex along the ventral medial prefrontal cortex in Fig. 2 of García-Cabezas and Barbas 2017). Abbreviations: AC, anterior commissure; cc, corpus callosum; Eulam., Eulaminate; Pir, piriform cortex; TP, temporal pole; Tt, tenia tecta. (A)–(D), modified from Uceda-Heras et al. (2024); (E), modified from García-Cabezas et al. (2020).

A critical point in cortical phylogenesis is that the cytoarchitectonically most complex cortical types, which are the primary sensory koniocortices, are identified in primates, but not in other mammals. This led Sanides (1962, 1970) to propose the hypothesis of the dual origin of the neocortex, which states that the tangential expansion of the primate cerebral cortex could be traced to two ancestral sectors in the allocortex: the ancestral hippocampal formation (aka archicortex) and the ancestral primary olfactory cortex (aka paleocortex). These two sectors formed a ring at the edge of each hemisphere. According to Sanides, the primate cerebral cortex expanded in successive concentric rings that developed inside the outer ancestral allocortical ring, starting from the mesocortical agranular (lacking a layer IV) areas into mesocortical dysgranular (with a rudimentary layer IV) areas and further into isocortical areas (which are eulaminate areas of six well-developed layers, including a granular layer IV) (Fig. 1A). Within isocortical areas, Sanides identified further concentric cortical types of progressively more elaborated laminar architecture, from the simplest isocortical areas to the most complex koniocortices. He concluded that, whereas allo- and mesocortical types could be observed across all mammals, the most complex isocortical types emerged later in evolution, having been identified exclusively in primates. The concentric arrangement of cortical types has been described in several mammalian species (Reep 1984) and is also identified in the highly expanded and “deformed” human cortex (Fig. 1B; see also García-Cabezas et al. 2023). Importantly, the concentric organization of cortical types along a gradient of laminar complexity, from limbic mesocortical areas, with a simple laminar structure, to koniocortical areas, exhibiting the most complex laminar structure, may be further split into two trends following the division of the ancestral outer allocortical ring: the hippocampal and olfactory sectors. Thus, these two ancestral sectors gave rise to two neocortical trends (parahippocampal and paraolfactory) as newer cortical areas developed in concentric inner neocortical rings, expanding the initial ring formed by the ancestral hippocampal formation and olfactory cortex (Fig. 2A-D).

Alternative hypotheses have been proposed to explain the tangential expansion of the primate cortex. Harvey Karten (1969) (see also Reiner 2000) proposed the hypothesis of the common origin of the temporal neocortex and the dorsal ventricular ridges (DVRs). According to Karten, the DVRs (two subcortical structures that form prominences on the surface of the lateral ventricle) of birds and reptiles and the temporal neocortex of mammals were homologs. The DVRs were considered part of the subpallium and Karten postulated a tangential migration from these ridges to the pallium. Contemporary developmental evidence does not support the ideas of Karten for two reasons. First, the DVRs in birds and reptiles are not subpallial; these structures and the pallial amygdala in mammals are homologs because they have common gene expression patterns and are placed topologically in equivalent sites of the telencephalon (Pessoa et al. 2019, Medina et al. 2023). Second, a massive tangential cell migration during cortical development from the DVR territory to the cortex has not been identified in any mammal. Later, Rakic (1995) proposed that the cortical expansion in primates could be the result of changes in the proliferation kinetics of progenitor founder cells that increase the number of radial columnar units: the more radial units, the larger the cortical surface. This proposal provided a cellular mechanism (the addition of new radial columnar units) for the tangential expansion of the primate cortex, which is consistent with Sanides’ proposal. Moreover, as it has been shown in macaque and human embryos (Reillo et al. 2011, Martinez-Cerdeno et al. 2012), the older mesocortical radial units may have less proliferative capacity compared to the newly added isocortical radial units that can generate abundant intermediate progenitors and basal radial glia cells. Finally, Luis Puelles and colleagues, by studying the patterns of gene expression in the developing cortex of mouse embryos and in adult mice (Puelles et al. 2019, Puelles et al. 2024), observed that allo-, meso-, and isocortical sectors were specified in concentric rings, as described by Sanides. However, Puelles et al. considered that Sanides’ trends may not be plausible because the entire mesocortex appeared to show a common genetic profile in adult mice, whereas the two paraolfactory and parahippocampal trends proposed by Sanides did no seem to show genetic differences. Thus, the entire mesocortex would have been formed along the whole border of the isocortex. Nonetheless, there are some morphogenetic genes that are expressed in the early pallium in gradients either from the hem (organizer adjacent to the medial pallium, which gives rise to the hippocampal allocortex) or from the anti-hem (organizer adjacent to the ventral pallium, which gives rise to the olfactory allocortex) into the prospective isocortex. More specifically, the expression of the genes *Left1* and *Lhx2* gradually decreases from the hem though the prospective hippocampal allocortex and parahippocampal mesocortex into the prospective isocortex; moreover, the expression of the gene *Tbr1* gradually decreases from the anti-hem in opposite direction. These overlapping gradients have been described in mouse, cat, and human embryos (Medina et al. 2004, Abellan et al. 2014, Gonzalez-Gomez and Meyer 2014, Siskos et al. 2021) and could result from gradients of diffusible morphogens secreted by both cortical organizers (hem and anti-hem; Subramanian et al. 2009). Thus, the genetic patterns observed by Puelles and colleagues in the adult mouse mesocortex could result from earlier specifications originated in the hem and anti-hem, which are adjacent to the ancestral allocortical moieties, and would promote a dual expansion of the neocortex, as first intuited by Sanides. Accordingly, the two organizers adjacent to the two ancestral allocortical sectors could give rise to the two neocortical trends (parahippocampal and paraolfactory).

Considering the dual origin hypothesis, limbic mesocortical areas, at one end of the neocortical laminar gradient and at high levels of the cortical hierarchy, are phylogenetically ancient, while better-laminated areas, at the other end of the gradient and at lower levels in the cortical hierarchy, are phylogenetically younger and emerged as inner rings. In primitive mammalian species, the cerebral cortex was generally undifferentiated, made of cortical types of simple architecture and polysensory in nature (Pandya et al. 2015). The phylogenetically younger types, which are exclusive to primates, enabled new levels in the cortical hierarchy, adding to the synaptic cortical circuits of primates relatively elaborate modality-specific processing and more computational capacity compared to the synaptic circuits of other mammals (Pandya et al. 2015), with phylogenetically older limbic mesocortical areas freed to play an increasingly sophisticated role of coordination and abstraction. This is supported by the observation that the degree of structural variation in the mouse cortex is far less pronounced than in the highly differentiated primate cortex (Fulcher et al. 2019); crucially, network density, defined as the number of existing connections divided by the number of potential connections, is much higher in the mouse than in primates, which suggests that, whereas in the mouse cortex almost all areas are connected with each other, in the primate cortex there are connective modules relatively isolated from each other (Markov et al. 2014, Ypma and Bullmore 2016; reviewed in Magrou et al. 2024). Further evidence is found in structure–function coupling, which has been shown to follow the cortical gradient of laminar complexity (Valk et al. 2022). In humans, lower-level unimodal cortical areas showed the highest structure–function coupling, whereas such coupling was lowest in limbic mesocortical areas (aka transmodal cortex). Interestingly, structure–function coupling analysis of macaque data revealed a similar distribution, but with increased coupling in association cortices relative to humans, thus, consistent with the hypothesis that the emergence of newer lower-level specialized cortices could enable functional changes, freeing higher-level areas to perform higher-level roles. Importantly, the possibility of functional evolution of phylogenetically preserved structures has been recently shown for the allocortex (hippocampus), which was demonstrated to have undergone functional adaptation despite preserved structure across macaques and humans (Eichert et al. 2024). Although how the allocortex (and subcortical areas) fits cortical hierarchies remains to be elucidated, as the structural model used to establish the direction of information flow (feedback or feedforward) applies only to neocortical areas [i.e. those having supra- (layers II and III) and infra- (layers V and VI) granular layers], evidence of functional evolution within phylogenetically ancient structures may contribute to explain the emergence of sophisticated forms of cognition.

Thus, the primitive polysensory role of limbic mesocortical areas may have evolved to a multimodal coordinating role within an ever more complex brain, favoring the possibilities of conscious experience. Furthermore, as we outline in the next section, some degree of functional specialization may have been preserved along the parahippocampal and paraolfactory trends derived from the two ancestral sectors.

## Two functional axes across cortical types

Following Sanides (1970), all neocortical areas may be placed within a gradient of laminar complexity as belonging to either the parahippocampal or the paraolfactory trend. When the areas contained in the two trends are analyzed, a potential rough functional distinction emerges. The parahippocampal and paraolfactory trends have been called “dorsal” and “ventral,” respectively, by Pandya et al. (2015), who proposed that the former was involved in object localization and operations in space, whereas the latter was involved in object analysis, facial expression, and verbalization-communication. We build on such work to substantiate such division more generally as navigation/spatial versus exchange/contact. Following Sanides’ original delimitation of the trends (Sanides 1970; represented in Fig. 2E), at the top of the neocortical hierarchy (i.e. the limbic mesocortical end of the laminar gradient) the parahippocampal trend comprises the cingulate, retrosplenial, prostriate, parahippocampal, and perirhinal cortices; the paraolfactory trend comprises the anterior insula (including what in nonhuman primates has been called motor proisocortex; Barbas and Pandya 1987), the temporal pole, and the posterior orbitofrontal cortex. From limbic mesocortical areas successive areas of both trends include progressively better laminated multimodal and unimodal association areas. Finally, at the lowest level of the cortical hierarchy, i.e. the other end of the laminar gradient, the parahippocampal trend includes the primary visual cortex and the dorsal part of the primary motor and primary somatosensory cortices, which include the representation of the lower limbs, the upper limbs and the trunk (Roux et al. 2018, Ghimire et al. 2021). On the other hand, the paraolfactory trend includes the primary auditory, gustatory, and interoceptive cortices, and the ventral part of the primary motor and primary somatosensory cortices, which include the representation of the head and neck, including face, mouth, tongue, and larynx representations (Roux et al. 2018, Ghimire et al. 2021). The motor and somatosensory representations of the hand are at the limit of both trends.

**Figure 3:**
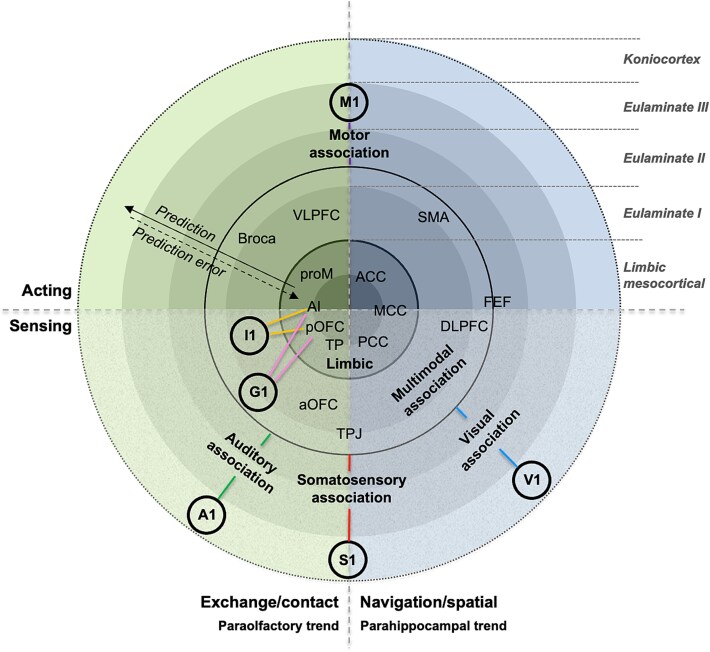
Two functional axes across cortical types in light of the dual origin hypothesis. The navigation versus exchange (horizontal) and acting versus sensing (vertical) axes define a conceptual bidimensional space across cortical types where virtually all cortical areas may be placed according to their functional relevance. Cortical types are represented as concentric circles, with limbic mesocortical areas at the center of the bidimensional space, sending predictions to outer circles: multimodal, unimodal association areas and ultimately primary cortices. Note that, to highlight the centrality of mesocortical limbic areas in cortical processing, the phylogenetically older rings are represented centrally and the newer rings are represented externally. Moreover, the illustration is conceived as schematic, and representative rather than exhaustive, without a strict spatial correspondence other than the different areas being placed on the corresponding quadrant and cortical type. Abbreviations: A1, primary auditory cortex; ACC, anterior cingulate cortex; AI, anterior insula; aOFC, anterior orbitofrontal cortex; DLPFC, dorsolateral prefrontal cortex; FEF, frontal eye field; G1, gustatory cortex; I1, interoceptive cortex; M1, primary motor cortex; MCC, mid cingulate cortex; PCC, posterior cingulate cortex; pOFC, posterior orbitofrontal cortex; proM, motor proisocortex; S1, primary somatosensory cortex; SMA, supplementary motor area; TP, temporal pole: TPJ, temporoparietal junction; V1, primary visual cortex; VLPFC, ventrolateral prefrontal cortex.

The division of body representation along somatosensory and motor cortices suggests that the parahippocampal and paraolfactory trends may roughly correspond to two different modes to approach the world: a navigation/spatial approach, and an intimate exchange/contact approach, respectively, constituting a first functional axis across cortical types relevant to allostasis and conscious experience (Fig. 3). In this context, the parahippocampal trend would be related to processing visual spatial information and to the execution of body movements related to spatial navigation in an open environment (legs, arms, and trunk). Indeed, the primate visual koniocortex may have emerged in evolution within the parahippocampal trend, being crucial to inform spatial navigation and musculoskeletal control. Interestingly, tract-tracing studies in macaques showed that areas along this trend issue projections to the pontine nuclei, in contrast with areas in the paraolfactory trend, which do not project or project sparsely to the pontine nuclei (reviewed in Schmahmann 2023). These nuclei are a key part of the cerebrocerebellar system that connects the cerebral cortex to the cerebellum in parallel cortico–pontine–cerebellar–thalamic–cortical loops that play a key role in the coordination of body movements and navigation (reviewed in Schmahmann 2023). It is also interesting to note that areas that have been shown to be “visceromotor,” such as de dorsal and subgenual anterior cingulate cortex (Kaada et al. 1949), developed in the parahippocampal trend, possibly due to their critical involvement in predictively supporting musculoskeletal needs (oxygen, glucose) for action. On the other hand, the paraolfactory trend would be related to auditory processing and language, head, and mouth movements, and to interoception and gustation, crucial for close interaction in the intimate and internal spheres, including stimuli inside the body through the mouth and bodily sensations (note that the primary olfactory cortex is in the olfactory allocortex, and thus connected to the paraolfactory trend as well). The auditory cortex, also paraolfactory, is key to interpersonal communication through spoken language, whose specific areas of Broca and Wernicke fall also within the paraolfactory trend.

A second functional axis across cortical types relevant to allostasis and conscious experience may be identified roughly based on sensory versus motor functions. Such functional distinction, which has been described in the classical works of Mesulam (1985, 1998), may be observed across cortical types, with primarily sensory or primarily motor areas being identified at all levels along the gradient of laminar complexity. For example, even at the limbic mesocortical level, where information is highly integrated and abstract, some areas may be considered more “motor,” such as the anterior cingulate cortex, whereas other areas may be considered more “sensory,” such as the posterior cingulate cortex. Moreover, we suggest that classically defined functions, such as working memory, mathematical operation, and so on, may be understood as “subsystems” or as sophisticated processes occurring at intermediate levels of a system that ultimately contributes roughly to sensing and acting, thus sustaining interaction with the world, allostasis, the organism’s wellbeing, and survival.

Together, the two axes summarized in the preceding paragraphs allow to place virtually all cortical areas on a bidimensional conceptual space that spans all neocortical types (Fig. 3). To note, such space and the two axes that define it are meant to be schematic and to function as a tool for interpretation and hypothesis generation, rather than a strictly direct mapping derived from empirical data.

Other authors have related the structural model and the dual origin hypothesis to predictive processing in the human brain. Notably, Tucker and Luu have discussed the topic in the context of control theory, using the terms “feedback” and “feedforward” with a different approach to the widely used meaning in the predictive processing literature: they refer to the limbipetal direction (from konio- through iso- into limbic mesocortices) as “feedback” and to the limbifugal direction (from limbic meso- through iso- into koniocortex) as “feedforward.” The authors justify these inversions of meaning because in control theory terms the sensory updating in the limbipetal direction is better described as feedback control. Tucker and Luu propose that the dorsal parahippocampal division of the limbic cortex issues primarily top-down limbifugal “feedforward” pathways that provide impulsive, endogenous impetus to action and generate expectations (predictions) during active inference; thus, this division would be linked to exploration. On the other hand, the ventral paraolfactory division of the limbic cortex issues limbipetal “feedback” pathways that provide constraint linked to exogenous events and generate error-correction during active inference; thus, this division is linked to environmental exploitation (Tucker and Luu 2023). Although these authors acknowledge that “feedforward” (limbifugal, top-down) and “feedback” (limbipetal, bottom-up) pathways are present along both neocortical trends, as stated by the structural model, they argue that “feedforward” expectations dominate in the dorsal parahippocampal trend, while “feedback” error corrections dominate in the ventral paraolfactory trend. More recently, Luu, Tucker, and Friston have related the different forms of cognitive control of the two limbic divisions and their neocortical trends to vertical integration of subcortical arousal control systems (Luu et al. 2024). The dorsal parahippocampal division is proposed to be regulated by phasic arousal, mediated by lemnothalamic pathways from the reticular activating system of the lower brainstem; the ventral paraolfactory division is proposed to be regulated by the tonic activation provided by collothalamic pathways from the midbrain arousal control centers. The regulation of the dorsal parahippocampal division is consolidated in REM sleep, while the regulation of the ventral paraolfactory division is consolidated in non-REM sleep (Tucker et al. 2025). In contrast to this proposal, we consider that feedback and feedforward co-occur along both cortical trends following the laminar gradient, while Tucker, Luu, and Friston attribute preferential “feedforward” and “feedback” roles to the dorsal parahippocampal and ventral paraolfactory trends, respectively. Moreover, we believe that the interpretation of collo- and lemnothalamic pathways does not encompass several subcortical thalamic afferents, like cerebellar and basal ganglia inputs to the ventral thalamic nuclei, whose cortical targets include frontal areas of both neocortical trends (reviewed in Barbas et al. 2013), retinal inputs to the lateral geniculate nucleus (Wiesel et al. 1974), and amygdalar afferents to the thalamic midline nuclei (Timbie et al. 2020); all these pathways can hardly be classified as collo- or lemnothalamic. High-resolution neuroimaging examining developmental trajectories and functional connectivity gradients in humans could provide further evidence for the proposed links between cortical types, dual-origin motifs, and higher-order cognitive functions.

## Implications for human experience

The two axes described in the previous section have important implications for our understanding of the neural basis of allostasis and conscious experience. Indeed, integration of the navigation-exchange and sensory-motor dimensions grounded along cortical types may be key to the emergence of a unified field of experience from the point of view of someone who has a body. It can be speculated that conscious experience could emerge as a consequence of increasingly sophisticated, adaptable bodies needing increasingly complex brains to regulate them; this could have led to the emergence of newer cortical types that could perform highly specialized functions, while phylogenetically older limbic mesocortical areas were freed from their polysensory role and could play an increasingly sophisticated role of coordination and abstraction.

Moreover, key large-scale brain networks supporting allostasis (default mode and salience networks; Kleckner et al. 2017, Zhang et al. 2023) span across eulaminate and mesocortical areas (see Fig. 1a in Paquola et al. 2025) along both neocortical trends in temporal, parietal, and frontal lobes. Thus, the default mode network would participate in the navigation/spatial versus exchange/contact and the sensory/motor axes, showing the relevance of highly abstract multimodal processes across such bidimensional space for the organism’s survival and well-being. The lateral connections (originated in both supra- and infragranular layers and terminating in all layers) between areas of the same cortical type may provide a bridge across the two sides of each axis in the default mode network, as well as other large-scale networks. Furthermore, although our model focuses on the cerebral cortex, subcortical structures play a fundamental role in processes such as action selection, reinforcement learning, and motor control. Integrating these subcortical dynamics with cortical predictive processing models could offer a more comprehensive account of how conscious experience emerges. We already mentioned that the parahippocampal trend participates in cerebrocerebellar circuits more than the paraolfactory trend (Schmahmann 2023). Regarding the basal ganglia, both cortical trends project to the striatum in an orderly manner according to cortical type (reviewed in Del Rey and García-Cabezas 2023) and the projections from each trend seem to have partially segregated territories (Yeterian and Pandya 1991). Moreover, thalamocortical and corticothalamic projections in primates also vary along the gradient of laminar complexity (Barbas and Zikopoulos 2025), thus, there could be specific thalamic territories for each cortical trend. Future studies may explore the interactions of the two cortical trends with basal ganglia, cerebellum, and thalamus to better grasp the richness of the neural underpinning of conscious experience.

The theoretical framework proposed in the present article has also important implications for our understanding of human pathology. It may function as a common framework to study the impact of focal brain lesions, as done in the above-mentioned seminal paper by Giaccio (2006). Moreover, the present proposal may also contribute to integrate different brain-related conditions into a common framework as related to disruptions of the general principles of brain structural and functional organization, further considering potential differences across evolutionary trends and the sensory-motor axis, even when appearing as highly abstract. For example, it could be speculated that the expansion of the cerebral cortex as described by Sanides could have pushed phylogenetically older limbic mesocortical areas, poor in protective cellular factors like myelin and perineuronal nets (García-Cabezas et al. 2017), to transition from a rough polysensory to a metabolically demanding coordinating role; this transition could have rendered them vulnerable to neurodegeneration (Bufill et al. 2013) and preferentially affected in mental-health-related conditions (Park et al. 2022). This conception may point at new paths to approach such conditions beyond the psychological sphere to include metabolism (e.g. Henkel et al. 2022) among other physiological processes.

## Conclusions

In the present article, we analyzed the integration of predictive processing approaches within the neuroanatomical notions of cortical gradient and cortical type, further grounding its evolutionary basis within the hypothesis of the dual origin of the cerebral cortex. By doing so, two axes with relevance for allostasis emerged, defining a conceptual bidimensional space across cortical types. Along these types, virtually all cortical areas may be placed according to their navigation-exchange and sensory-motor relevance. Such space may contribute to integrate knowledge on the neural basis of conscious experience and brain-related conditions, in a common framework with the potential to reveal new insights beyond the psychological sphere and the brain.

## Data Availability

There is no new data associated with this article.

## References

[ref1] Abellan A, Desfilis E, Medina L. Combinatorial expression of Lef1, Lhx2, Lhx5, Lhx9, Lmo3, Lmo4, and Prox1 helps to identify comparable subdivisions in the developing hippocampal formation of mouse and chicken. *Front Neuroanat* 2014;8:59. 10.3389/fnana.2014.00059 25071464 PMC4082316

[ref2] An X, Bandler R, Ongur D et al. Prefrontal cortical projections to longitudinal columns in the midbrain periaqueductal gray in macaque monkeys. *J Comp Neurol* 1998;401:455–79. 9826273

[ref3] Aparicio-Rodríguez G, García-Cabezas MÁ. Comparison of the predictive power of two models of cortico-cortical connections in primates: the distance rule model and the structural model. *Cereb Cortex* 2023;33:8131–49. 10.1093/cercor/bhad104 37041104

[ref4] Barbas H . General cortical and special prefrontal connections: principles from structure to function. *Annu Rev Neurosci* 2015;38:269–89. 10.1146/annurev-neuro-071714-033936 25897871

[ref5] Barbas H, Blatt GJ. Topographically specific hippocampal projections target functionally distinct prefrontal areas in the rhesus monkey. *Hippocampus* 1995;5:511–33. 10.1002/hipo.450050604 8646279

[ref6] Barbas H, Pandya DN. Architecture and frontal cortical connections of the premotor cortex (area 6) in the rhesus monkey. *J Comp Neurol* 1987;256:211–28. 3558879 10.1002/cne.902560203

[ref7] Barbas H, Rempel-Clower N. Cortical structure predicts the pattern of corticocortical connections. *Cereb Cortex* 1997;7:635–46. 9373019 10.1093/cercor/7.7.635

[ref8] Barbas H, Zikopoulos B. The cortical structural model extends to thalamocortical connections. *Eur J Neurosci* 2025;61:e70167. 10.1111/ejn.70167 40542691 PMC12723798

[ref9] Barbas H, Garcia-Cabezas MA, Zikopoulos B. Frontal-thalamic circuits associated with language. *Brain Lang* 2013;126:49–61. 10.1016/j.bandl.2012.10.001 23211411 PMC3615046

[ref10] Barbas H, Garcia-Cabezas MA, John Y et al. Cortical circuit principles predict patterns of trauma induced tauopathy in humans. *Cer Cor* 2025;35:bhaf209. 10.1093/cercor/bhaf209 PMC12357493 40817910

[ref11] Barrett LF . How Emotions Are Made: The Secret Life of the Brain. Boston: Houghton Mifflin Harcourt, 2017.

[ref12] Barrett LF, Simmons WK. Interoceptive predictions in the brain. *Nat Rev Neurosci* 2015;16:419–29. 10.1038/nrn3950 26016744 PMC4731102

[ref13] Bastos AM, Usrey WM, Adams RA et al. Canonical microcircuits for predictive coding. *Neuron* 2012;76:695–711. 10.1016/j.neuron.2012.10.038 23177956 PMC3777738

[ref14] Bufill E, Blesa R, Augusti J. Alzheimer’s disease: an evolutionary approach. *J Anthropol Sci* 2013;91:135–57. 10.4436/jass.91001 23579031

[ref15] Carmichael ST, Clugnet MC, Price JL. Central olfactory connections in the macaque monkey. *J Comp Neurol* 1994;346:403–34. 10.1002/cne.903460306 7527806

[ref16] Chanes L, Barrett LF. Redefining the role of limbic areas in cortical processing. *Trends Cogn Sci* 2016;20:96–106. 10.1016/j.tics.2015.11.005 26704857 PMC4780414

[ref17] Chanes L, Barrett LF. The predictive brain, conscious experience, and brain-related conditions. In: Mendonça D, Curado M, Gouveia SS (eds.), *The Philosophy and Science of Predictive Processing*, pp. 159–69, Bloomsbury Academic, London, 2020.

[ref18] Clark A . Whatever next? Predictive brains, situated agents, and the future of cognitive science. *Behav Brain Sci* 2013;36:181–204. 10.1017/S0140525X12000477 23663408

[ref19] Dehaene S, Changeux JP. Experimental and theoretical approaches to conscious processing. *Neuron* 2011;70:200–27. 10.1016/j.neuron.2011.03.018 21521609

[ref20] Del Rey NL, García-Cabezas MÁ. Cytology, architecture, development, and connections of the primate striatum: hints for human pathology. *Neurobiol Dis* 2023;176:105945. 10.1016/j.nbd.2022.105945 36481436

[ref21] Eichert N, DeKraker J, Howard AFD et al. Hippocampal connectivity patterns echo macroscale cortical evolution in the primate brain. *Nat Commun* 2024;15:5963. 10.1038/s41467-024-49823-8 39013855 PMC11252401

[ref22] Friston K . A theory of cortical responses. *Philos Trans R Soc Lond Ser B Biol Sci* 2005;360:815–36. 10.1098/rstb.2005.1622 15937014 PMC1569488

[ref23] Friston K . The free-energy principle: a unified brain theory? *Nat Rev Neurosci* 2010;11:127–38. 10.1038/nrn2787 20068583

[ref24] Fulcher BD, Murray JD, Zerbi V et al. Multimodal gradients across mouse cortex. *Proc Natl Acad Sci U S A* 2019;116:4689–95. 10.1073/pnas.1814144116 30782826 PMC6410879

[ref25] García-Cabezas MÁ, Barbas H. Anterior cingulate pathways may affect emotions through orbitofrontal cortex. *Cereb Cortex* 2017;27:4891–910. 10.1093/cercor/bhw284 27655930 PMC6075591

[ref26] García-Cabezas MÁ, Joyce MKP, John YJ et al. Mirror trends of plasticity and stability indicators in primate prefrontal cortex. *Eur J Neurosci* 2017;46:2392–405. 10.1111/ejn.13706 28921934 PMC5656436

[ref27] García-Cabezas MÁ, Barbas H, Zikopoulos B. Parallel development of chromatin patterns, neuron morphology, and connections: potential for disruption in autism. *Front Neuroanat* 2018;12:70. 10.3389/fnana.2018.00070 30174592 PMC6107687

[ref28] García-Cabezas MÁ, Zikopoulos B, Barbas H. The structural model: a theory linking connections, plasticity, pathology, development and evolution of the cerebral cortex. *Brain Struct Funct* 2019;224:985–1008. 10.1007/s00429-019-01841-9 30739157 PMC6500485

[ref29] García-Cabezas MÁ, Hacker JL, Zikopoulos B. A protocol for cortical type analysis of the human neocortex applied on histological samples, the Atlas of Von Economo and Koskinas, and magnetic resonance imaging. *Front Neuroanat* 2020;14:576015. 10.3389/fnana.2020.576015 33364924 PMC7750391

[ref30] García-Cabezas MÁ, Hacker JL, Zikopoulos B. Homology of neocortical areas in rats and primates based on cortical type analysis: an update of the hypothesis on the dual origin of the neocortex. *Brain Struct Funct* 2023;228:1069–93. 10.1007/s00429-022-02548-0 35962240 PMC9922339

[ref31] Ghashghaei HT, Hilgetag CC, Barbas H. Sequence of information processing for emotions based on the anatomic dialogue between prefrontal cortex and amygdala. *Neuroimage* 2007;34:905–23. 10.1016/j.neuroimage.2006.09.046 17126037 PMC2045074

[ref32] Ghimire P, Lavrador JP, Baig Mirza A et al. Intraoperative mapping of pre-central motor cortex and subcortex: a proposal for supplemental cortical and novel subcortical maps to Penfield’s motor homunculus. *Brain Struct Funct* 2021;226:1601–11. 10.1007/s00429-021-02274-z 33871691 PMC8096772

[ref33] Giaccio RG . The dual origin hypothesis: an evolutionary brain-behavior framework for analyzing psychiatric disorders. *Neurosci Biobehav Rev* 2006;30:526–50. 10.1016/j.neubiorev.2005.04.021 16356547

[ref34] Gonzalez-Gomez M, Meyer G. Dynamic expression of calretinin in embryonic and early fetal human cortex. *Front Neuroanat* 2014;8:41. 10.3389/fnana.2014.00041 24917793 PMC4042362

[ref35] Henkel ND, Wu X, O'Donovan SM et al. Schizophrenia: a disorder of broken brain bioenergetics. *Mol Psychiatry* 2022;27:2393–404. 10.1038/s41380-022-01494-x 35264726

[ref36] Kaada BR, Pribram KH, Epstein JA. Respiratory and vascular responses in monkeys from temporal pole, insula, orbital surface and cingulate gyrus; a preliminary report. *J Neurophysiol* 1949;12:347–56. 10.1152/jn.1949.12.5.347 18137711

[ref37] Karten HJ . The organization of the avian telencephalon and some speculations on the phylogeny of the amniote telencephalon. *Ann N Y Acad Sci* 1969;167:164–79.

[ref38] Katsumi Y, Theriault JE, Quigley KS et al. Allostasis as a core feature of hierarchical gradients in the human brain. *Netw Neurosci* 2022;6:1010–31. 10.1162/netn_a_00240 38800458 PMC11117115

[ref39] Katsumi Y, Zhang J, Chen D et al. Correspondence of functional connectivity gradients across human isocortex, cerebellum, and hippocampus. *Commun Biol* 2023;6:401. 10.1038/s42003-023-04796-0 37046050 PMC10097701

[ref40] Kiebel SJ, Daunizeau J, Friston KJ. A hierarchy of time-scales and the brain. *PLoS Comput Biol* 2008;4:e1000209. 10.1371/journal.pcbi.1000209 19008936 PMC2568860

[ref41] King L, Weiner KS. Transcriptomic contributions to a modern cytoarchitectonic parcellation of the human cerebral cortex. *Brain Struct Funct* 2024;229:919–36. 10.1007/s00429-023-02754-4 38492042

[ref42] Kleckner IR, Zhang J, Touroutoglou A et al. Evidence for a large-scale brain system supporting allostasis and interoception in humans. *Nat Hum Behav* 2017;1:0069. 10.1038/s41562-017-0069 28983518 PMC5624222

[ref43] Luu P, Tucker DM, Friston K. From active affordance to active inference: vertical integration of cognition in the cerebral cortex through dual subcortical control systems. *Cereb Cortex* 2024;34:bhad458. 10.1093/cercor/bhad458 38044461

[ref44] Magrou L, Joyce MKP, Froudist-Walsh S et al. The meso-connectomes of mouse, marmoset, and macaque: network organization and the emergence of higher cognition. *Cereb Cortex* 2024;34:bhae174. 10.1093/cercor/bhae174 38771244 PMC11107384

[ref45] Margulies DS, Ghosh SS, Goulas A et al. Situating the default-mode network along a principal gradient of macroscale cortical organization. *Proc Natl Acad Sci U S A* 2016;113:12574–9. 10.1073/pnas.1608282113 27791099 PMC5098630

[ref46] Markov NT, Ercsey-Ravasz MM, Ribeiro Gomes AR et al. A weighted and directed interareal connectivity matrix for macaque cerebral cortex. *Cereb Cortex* 2014;24:17–36. 10.1093/cercor/bhs270 23010748 PMC3862262

[ref47] Martinez-Cerdeno V, Cunningham CL, Camacho J et al. Comparative analysis of the subventricular zone in rat, ferret and macaque: evidence for an outer subventricular zone in rodents. *PLoS One* 2012;7:e30178. 10.1371/journal.pone.0030178 22272298 PMC3260244

[ref48] Mashour GA, Roelfsema P, Changeux JP et al. Conscious processing and the global neuronal workspace hypothesis. *Neuron* 2020;105:776–98. 10.1016/j.neuron.2020.01.026 32135090 PMC8770991

[ref49] Medina L, Legaz I, Gonzalez G et al. Expression of Dbx1, Neurogenin 2, Semaphorin 5A, cadherin 8, and Emx1 distinguish ventral and lateral pallial histogenetic divisions in the developing mouse claustroamygdaloid complex. *J Comp Neurol* 2004;474:504–23. 10.1002/cne.20141 15174069

[ref50] Medina L, Abellan A, Morales L et al. Evolution and development of amygdala subdivisions: pallial, subpallial, and beyond. *Brain Behav Evol* 2023;98:1–21. 10.1159/000527512 36265454

[ref51] Mesulam MM . Patterns in behavioral neuroanatomy: association areas, the limbic system, and hemispheric specialization. In: Mesulam MM (ed.), *Principles of Behavioral Neurology*, pp. 1–70. Philadelphia: F.A. Davis Company, 1985.

[ref52] Mesulam MM . From sensation to cognition. *Brain* 1998;121(Pt 6):1013–52. 9648540 10.1093/brain/121.6.1013

[ref53] Murray JD, Bernacchia A, Freedman DJ et al. A hierarchy of intrinsic timescales across primate cortex. *Nat Neurosci* 2014;17:1661–3. 10.1038/nn.3862 25383900 PMC4241138

[ref54] Ohm DT, Xie SX, Capp N et al. Cytoarchitectonic gradients of laminar degeneration in behavioural variant frontotemporal dementia. *Brain* 2024;148:102–18. 10.1093/brain/awae263 PMC11706280 39119853

[ref55] Ottoy J, Kang MS, Tan JXM et al. Tau follows principal axes of functional and structural brain organization in Alzheimer’s disease. *Nat Commun* 2024;15:5031. 10.1038/s41467-024-49300-2 38866759 PMC11169286

[ref56] Pandya DN, Seltzer B, Petrides M et al. Cerebral Cortex: Architecture, Connections, and the Dual Origin Concept. Oxford: Oxford University Press, 2015.

[ref57] Paquola C, Amunts K, Evans A et al. Closing the mechanistic gap: the value of microarchitecture in understanding cognitive networks. *Trends Cogn Sci* 2022;26:873–86. 10.1016/j.tics.2022.07.001 35909021

[ref58] Paquola C, Garber M, Frassle S et al. The architecture of the human default mode network explored through cytoarchitecture, wiring and signal flow. *Nat Neurosci* 2025;28:654–64. 10.1038/s41593-024-01868-0 39875581 PMC11893468

[ref59] Park BY, Kebets V, Lariviere S et al. Multiscale neural gradients reflect transdiagnostic effects of major psychiatric conditions on cortical morphology. *Commun Biol* 2022;5:1024. 10.1038/s42003-022-03963-z 36168040 PMC9515219

[ref60] Parkes L, Kim JZ, Stiso J et al. Asymmetric signaling across the hierarchy of cytoarchitecture within the human connectome. *Sci Adv* 2022;8:eadd2185. 10.1126/sciadv.add2185 36516263 PMC9750154

[ref61] Pessoa L, Medina L, Hof PR et al. Neural architecture of the vertebrate brain: implications for the interaction between emotion and cognition. *Neurosci Biobehav Rev* 2019;107:296–312. 10.1016/j.neubiorev.2019.09.021 31541638 PMC6996540

[ref62] Puelles L, Alonso A, Garcia-Calero E et al. Concentric ring topology of mammalian cortical sectors and relevance for patterning studies. *J Comp Neurol* 2019;527:1731–52. 10.1002/cne.24650 30737959

[ref63] Puelles L, Alonso A, Garcia-Calero E. Genoarchitectural definition of the adult mouse mesocortical ring: a contribution to cortical ring theory. *J Comp Neurol* 2024;532:e25647. 10.1002/cne.25647 38961708

[ref64] Rakic P . A small step for the cell, a giant leap for mankind: a hypothesis of neocortical expansion during evolution. *Trends Neurosci* 1995;18:383–8. 10.1016/0166-2236(95)93934-p 7482803

[ref65] Reep R . Relationship between prefrontal and limbic cortex: a comparative anatomical review. *Brain Behav Evol* 1984;25:5–80. 10.1159/000118849 6398115

[ref66] Reillo I, Romero CD, García-Cabezas MA et al. A role for intermediate radial glia in the tangential expansion of the mammalian cerebral cortex. *Cereb Cortex* 2011;21:1674–94. 10.1093/cercor/bhq238 21127018

[ref67] Reiner AJ . A hypothesis as to the organization of cerebral cortex in the common amniote ancestor of modern reptiles and mammals. *Novartis Found Symp* 2000;228:83–102 discussion 102–113. 10.1002/0470846631.ch7 10929318

[ref68] Rempel-Clower NL, Barbas H. Topographic organization of connections between the hypothalamus and prefrontal cortex in the rhesus monkey. *J Comp Neurol* 1998;398:393–419. 10.1002/(sici)1096-9861(19980831)398:3<393::aid-cne7>3.0.co;2-v 9714151

[ref69] Roux FE, Djidjeli I, Durand JB. Functional architecture of the somatosensory homunculus detected by electrostimulation. *J Physiol* 2018;596:941–56. 10.1113/JP275243 29285773 PMC5830421

[ref70] Ruiz-Cabrera S, Pérez-Santos I, Zaldivar-Diez J et al. Expansion modes of primate nervous system structures in the light of the prosomeric model. *Front Mamm Sci* 2023;2:1241573. 10.3389/fmamm.2023.1241573

[ref71] Saberi A, Paquola C, Wagstyl K et al. The regional variation of laminar thickness in the human isocortex is related to cortical hierarchy and interregional connectivity. *PLoS Biol* 2023;21:e3002365. 10.1371/journal.pbio.3002365 37943873 PMC10684102

[ref72] Sancha-Velasco A, Uceda-Heras A, García-Cabezas MÁ. Cortical type: a conceptual tool for meaningful biological interpretation of high-throughput gene expression data in the human cerebral cortex. *Front Neuroanat* 2023;17:1187280. 10.3389/fnana.2023.1187280 37426901 PMC10323436

[ref73] Sanides F . Architectonics of the human frontal lobe of the brain. With a demonstration of the principles of its formation as a reflection of phylogenetic differentiation of the cerebral cortex. *Monogr Gesamtgeb Neurol Psychiatr* 1962;98:1–201. 13976313

[ref74] Sanides F . Functional architecture of motor and sensory cortices in primates in the light of a new concept of neocortex evolution. In: Noback CR, Montagna W (eds.), *The Primate Brain: Advances in Primatology*, pp. 137–208. New York (NY): Appleton-Century-Crofts Educational Division/Meredith Corporation, 1970.

[ref75] Schmahmann JD . Chapter 11, the cerebrocerebellar system. In: Gruol DL, Koibuchi N, Manto M et al. (eds.), Essentials of Cerebellum and Cerebellar Disorders. Cham, Switzerland: Springer, 2023, 77–86. 10.1007/978-3-031-15070-8.

[ref76] Sebenius I, Dorfschmidt L, Seidlitz J et al. Structural MRI of brain similarity networks. *Nat Rev Neurosci* 2024;26:42–59. 10.1038/s41583-024-00882-2 39609622 PMC12936990

[ref77] Siskos N, Ververidis C, Skavdis G et al. Genoarchitectonic compartmentalization of the embryonic telencephalon: insights from the domestic cat. *Front Neuroanat* 2021;15:785541. 10.3389/fnana.2021.785541 34975420 PMC8716433

[ref78] Subramanian L, Remedios R, Shetty A et al. Signals from the edges: the cortical hem and antihem in telencephalic development. *Semin Cell Dev Biol* 2009;20:712–8. 10.1016/j.semcdb.2009.04.001 19446478 PMC2791850

[ref79] Timbie C, García-Cabezas MÁ, Zikopoulos B et al. Organization of primate amygdalar-thalamic pathways for emotions. *PLoS Biol* 2020;18:e3000639. 10.1371/journal.pbio.3000639 32106269 PMC7064256

[ref80] Tucker DM, Luu P. Adaptive control of functional connectivity: dorsal and ventral limbic divisions regulate the dorsal and ventral neocortical networks. *Cereb Cortex* 2023;33:7870–95. 10.1093/cercor/bhad085 36958794

[ref81] Tucker DM, Luu P, Friston KJ. Adaptive consolidation of active inference: excitatory and inhibitory mechanisms for organizing feedforward and feedback memory systems in sleep. *Cereb Cortex* 2025;35:bhaf122. 10.1093/cercor/bhaf122 40422982 PMC13017609

[ref82] Uceda-Heras A, Aparicio-Rodriguez G, Garcia-Cabezas MA. Hyperphosphorylated tau in Alzheimer’s disease disseminates along pathways predicted by the structural model for cortico-cortical connections. *J Comp Neurol* 2024;532:e25623. 10.1002/cne.25623 38803103

[ref83] Valk SL, Xu T, Paquola C et al. Genetic and phylogenetic uncoupling of structure and function in human transmodal cortex. *Nat Commun* 2022;13:2341. 10.1038/s41467-022-29886-1 35534454 PMC9085871

[ref84] Weber CF, Kebets V, Benkarim O et al. Contracted functional connectivity profiles in autism. *Mol Autism* 2024;15:38. 10.1186/s13229-024-00616-2 39261969 PMC11391747

[ref85] Wiesel TN, Hubel DH, Lam DM. Autoradiographic demonstration of ocular-dominance columns in the monkey striate cortex by means of transneuronal transport. *Brain Res* 1974;79:273–9. 10.1016/0006-8993(74)90416-8 4423575

[ref86] Yeterian EH, Pandya DN. Prefrontostriatal connections in relation to cortical architectonic organization in rhesus monkeys. *J Comp Neurol* 1991;312:43–67. 10.1002/cne.903120105 1744243

[ref87] Ypma RJ, Bullmore ET. Statistical analysis of tract-tracing experiments demonstrates a dense, complex cortical network in the mouse. *PLoS Comput Biol* 2016;12:e1005104. 10.1371/journal.pcbi.1005104 27617835 PMC5019374

[ref88] Zhang J, Scholtens LH, Wei Y et al. Topography impacts topology: anatomically central areas exhibit a “high-level connector” profile in the human cortex. *Cereb Cortex* 2020;30:1357–65. 10.1093/cercor/bhz171 31504277 PMC7132940

[ref89] Zhang J, Chen D, Srirangarajan T et al. Cortical and subcortical mapping of the allostatic-interoceptive system in the human brain: replication and extension with 7 Tesla fMRI. bioRxiv. 2023. 10.1101/2023.07.20.548178 PMC12586188 41131362

[ref90] Zikopoulos B, Barbas H. Changes in prefrontal axons may disrupt the network in autism. *J Neurosci* 2010;30:14595–609. 10.1523/JNEUROSCI.2257-10.2010 21048117 PMC3073590

[ref91] Zikopoulos B, García-Cabezas MA, Barbas H. Parallel trends in cortical gray and white matter architecture and connections in primates allow fine study of pathways in humans and reveal network disruptions in autism. *PLoS Biol* 2018;16:e2004559. 10.1371/journal.pbio.2004559 29401206 PMC5814101

